# Investigation depression prevalence and related effective factors among students at health faculty isfahan university 2019, Iran

**DOI:** 10.1192/j.eurpsy.2021.885

**Published:** 2021-08-13

**Authors:** M. Aboutalebi, G. Mehvari, E. Heidari

**Affiliations:** 1 Psychiatry, University, Isfahan, Iran; 2 Psychology, Isfahan University, Isfahan, Iran

**Keywords:** Student, Depression, Beak test, Isfahan

## Abstract

**Introduction:**

The incidence of depression is associated with decreased social, occupational, and educational performance.

**Objectives:**

The aim of this study was assessing the prevalence of depression and its related effective factors among students at health faculty at Isfahan University of Medical Sciences in 2019.

**Methods:**

In this cross-sectional study 177 students were included randomly. The Beak test included 21 questions were applied to collect data. Data were analyzed by SPSS software (version 22) and were presented as descriptive statistics and analyses included One-way analysis of variance, t-test and correlation Pearson.

**Results:**

The mean and standard deviation of the age of students was 22.15±3.88 years. More than 80% of students experienced some levels of depression. Of the participants 19.8% indicated no sign of distress, 26% mild distress, 37.3% average distress and 16.9% high depression. There was no statistical association of distress between female and male students (P=0.198). However, significant associations were Sedative drugs, parents level and occupation, Study Field, Future Career and Financial situation with depression (P<0.05).

**Conclusions:**

Overall, the prevalence of depression was higher among students compared with general population. Providing programs for improving student’s mental health is suggested.

